# Comparison of anticoagulated versus non-anticoagulated patients with intra-aortic balloon pumps

**DOI:** 10.1186/s12959-021-00295-6

**Published:** 2021-06-29

**Authors:** Julie Kelly, Rhynn Malloy, Danielle Knowles

**Affiliations:** Department of Pharmacy Services, Brigham and Women’s Hospital, 75 Francis St Boston, Boston, MA 02215 USA

**Keywords:** Intra-aortic balloon pumps, Anticoagulation, Mechanical circulatory support

## Abstract

**Background:**

There is limited guidance regarding the use of anticoagulation in patients on intra-aortic balloon pumps (IABP). The purpose of this study is to compare the safety outcomes in anticoagulated versus non-anticoagulated patients with an IABP.

**Methods:**

This was a single center, retrospective chart review of patients admitted to the coronary care unit or cardiac surgery unit who received an IABP from May 2015 to July 2018. Patients who were anticoagulated with heparin while on an IABP were compared to those who were not anticoagulated. Major endpoints included a composite of thrombotic events and a composite of bleeding events. The major composite endpoint of thrombotic events included the incidence of ischemic stroke, any venous thromboembolism, device thrombosis, and limb ischemia. The major composite endpoint of bleeding events included major access site bleeding, minor access site bleeding, major non-access site bleeding, and minor non-access site bleeding. Minor endpoints included any major endpoint events occurring within 24 and 48 h of IABP insertion, hospital length of stay, intensive care unit length of stay, and in-hospital mortality.

**Results:**

A total of 185 patients were evaluated for inclusion and 147 were included in the final analysis. There were 82 and 65 patients in the heparin and non-heparin groups, respectively. The composite endpoint of thrombotic events occurred in 7.3 and 7.7% in the heparin and non-heparin groups, respectively (*p* = 1). The composite bleeding endpoint occurred in 20.7 and 20.0% in the heparin and non-heparin groups, respectively (*p* = 0.91). There were no differences found in minor endpoints between groups.

**Conclusion:**

There were no significant differences found in major endpoints of bleeding and thrombotic events in patients who received anticoagulation while on an IABP versus those who did not receive anticoagulation.

## Introduction

Intra-aortic balloon pumps (IABPs) are a form of mechanical circulatory support used to enhance cardiac output and increase coronary artery perfusion. Heparin is often used in patients with an IABP, as early registry data have shown an increased risk of limb ischemia and other thrombotic events [1]. Due to the risk of platelet shearing and platelet adhesion to the IABP membrane, thrombocytopenia is a common complication that leads to an increased risk of bleeding [1–3]. The bleeding and thrombotic risks of IABP placement in addition to the limited definitive data make it difficult to determine an optimal anticoagulation strategy. Current mechanical circulatory support guidelines make a brief, general statement that each institution should establish their own protocol on anticoagulation in IABPs based on patient specific risk factors [4].

There is a lack of data available to provide guidance on whether patients with an IABP should be anticoagulated. One animal trial randomized 25 pigs to either systemic heparin, heparin-bound IABP, or no heparin. The IABPs were examined for thrombus, and there was no evidence of thrombus in either heparin group, however there was statistically more device thrombosis found in the non-heparin group [5]. In addition to animal studies, a small randomized trial and a cohort study evaluated the use of anticoagulation in patients with an IABP with variable patient populations and inconsistent results [6, 7].

Brigham and Women’s Hospital uses IABPs across multiple services, including the coronary care unit (CCU) and the cardiac surgery unit (CSICU). In the CCU, it is common practice to anticoagulate patients on an IABP, typically using heparin with a partial thromboplastin time (PTT) goal of 60–80 s. The CSICU does not typically utilize anticoagulation by either infusion or bolus unless they are on an assist ratio of 1:2 to 1:3 for greater than 30 min. Our institution utilizes non-heparin coated Datascope© manufactured IABPs size 30 to 50 cm. CSICU patients typically have IABPs placed in the operating room (OR). IABP placement in CCU patients typically occurs in the catheterization lab, however this can vary based on clinic scenario. The purpose of this study was to provide further insight into the need for anticoagulation in IABPs by comparing bleeding and thrombotic outcomes in a non-anticoagulated versus anticoagulated population of patients with an IABP.

## Methods

This was a single center, retrospective study at a tertiary academic medical center, approved by the Partner’s Healthcare Institutional Review Board. All data were obtained from electronic medical records.

Patients were included if they were admitted to the CCU or CSICU from June 2015 to July 2018 with an IABP placed at any time during the admission. Patients were excluded if they were placed on if they were on an anticoagulant other than unfractionated heparin while on an IABP, or if they received an IABP after an endarterectomy procedure. Patients who received therapeutic unfractionated heparin (UFH) were compared to those who did not receive anticoagulation. Additionally, a subgroup analysis was performed excluding patients who were on anticoagulation at home prior to hospitalization. This was done to specifically examine patients who did not have another indication for anticoagulation other than the IABP device.

The major safety endpoint included a composite of major and minor non-access site bleeding, and major and minor access site bleeding, both defined by the International Society on Thrombosis and Haemostasis (ISTH) [8]. The major endpoint of thrombotic events included a composite of incidence of ischemic stroke, any venous thromboembolism, device thrombosis, and limb ischemia. Bleeding and thrombotic events were collected from the time of IABP insertion until the end of the indexed hospitalization. Minor endpoints included major endpoint events occurring within 24 and 48 h of IABP insertion, hospital length of stay, intensive care unit length of stay, and in-hospital mortality. Time on heparin, PTT goal, and time in goal PTT range were collected for the heparin group in addition to the concomitant use of aspirin and dual antiplatelet therapy (DAPT) with aspirin and a P2Y_12_ inhibiter.

Continuous data were compared using t-test for parametric data and Mann Whitney U statistical testing for non-parametric data as appropriate. Categorical data were compared using Chi-Square test and Fisher’s Exact test as appropriate. Statistical significance was set at a level of *p* < 0.05. In addition, a post-hoc analysis compared major and minor outcomes between the two groups using a logistic regression multivariate analysis adjusted for sex, creatinine clearance, use of DAPT, and patient’s location (CCU versus CSICU). Statistical analyses were performed using SPSS and STATA software.

## Results

**Fig. 1:**
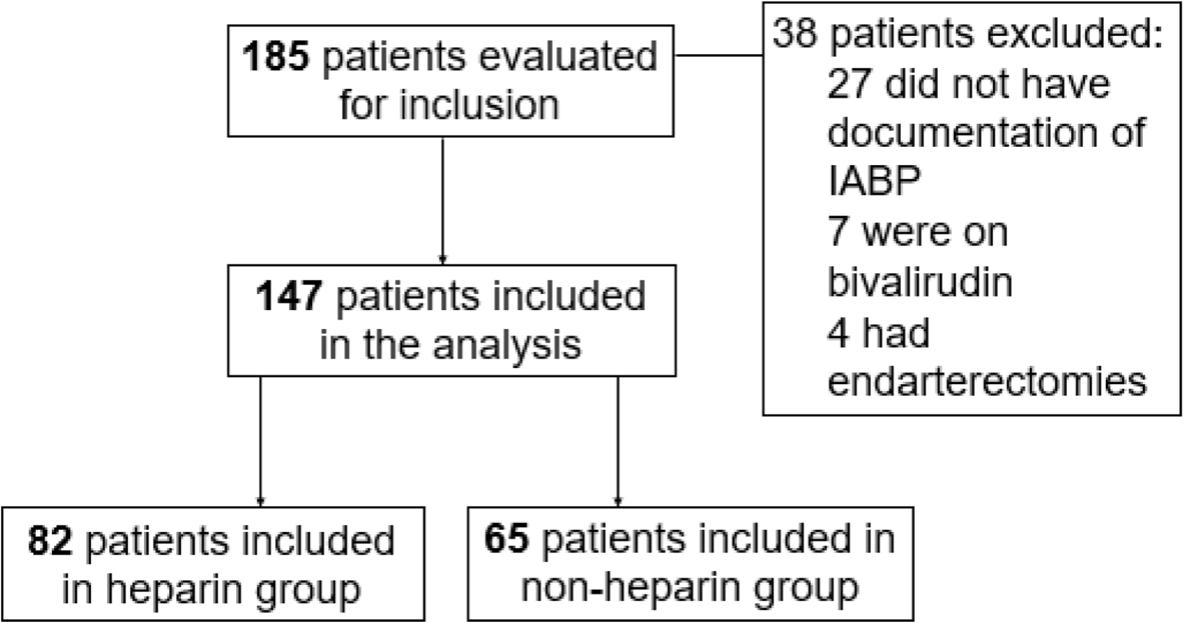
Patient Enrollment. ECMO = extracorpeal membrane oxygenation

Of the 185 patient charts that were evaluated for inclusion, 147 patients were included in the final analysis. A total of 82 patients were included in the heparin group and 65 patients in the non-heparin group (see Fig. 1).

Patient baseline characteristics were similar between the two groups with the exception of a lower incidence of baseline valvular disease which was lower in the heparin group as compared to the non-heparin group (15.9% vs 36.9%) (Table 1). The majority of heparinized patients were admitted to the CCU (58.5%) while the majority of non-heparinized patients were admitted to the CSICU (83.1%).

**Table 1:** Baseline Characteristics

	Heparin group (*n* = 82)	Non-heparin group (*n* = 65)
Age (years)^a^	67 ± 10.8	68 ± 12.1
Sex (male)	52 (63.4)	51 (78.5)
Body mass index (kg/m^2^)^a^	29.3 ± 6.3	29.2 ± 7.5
Creatinine clearance (mL/min)^a^	67.2 ± 31.3	62.7 ± 34.5
Admitted to coronary care unit	48 (58.5)	11 (16.9)
Admitted to cardiac surgery	34 (41.5)	53 (83.1)
Hypertension	58 (70.7)	55 (84.6)
Atrial arrhythmias	18 (22.0)	14 (21.5)
Ventricular arrhythmias	10 (12.2)	8 (12.3)
Valvular disease	13 (15.9)	24 (36.9)
Coronary artery disease	47 (72.3)	39 (60.0)
Heart failure	25 (30.5)	21 (32.3)
On concurrent extracorpeal membrane oxygenation	10 (12.2)	1 (1.5)

All values are n(%) unless otherwise specified

^a^mean ± standard deviation

The heparin group had more patients with a primary diagnosis of ST-elevation myocardial infarction (STEMI) as compared to the non-heparin group (22% vs 4.6%, *p* < 0.01), while there were fewer patients with a primary diagnosis of coronary artery bypass graft (CABG) as compared to the non-heparin group (14.6% vs 56.9%, *p* < 0.01) (Table 2). Patients in the heparin group had a higher median time on an IABP as compared to the non-heparin group (42 h; [IQR, 30–68] vs 26 h; [IQR, 17–45], *p* < 0.01) with a longer median time in the 1:1 assist ratio setting as compared to the non-heparin group (36 h; [IQR, 18.8–62.5] vs 19 h; [IQR, 10–40], *p* < 0.01). Time in the 1:2 and 1:3 assist ratios was similar between the two groups. The majority of patients in the heparin group had a documented goal PTT of 60–80 s (65.9%) and patients were in goal range 44.8% of the time on average (Table 3). There was no difference in the concomitant use of aspirin monotherapy between the heparin group and non-heparin group (Table 3). There were more patients in the heparin group on DAPT (26.8% vs 13.8%, *p* < 0.03).

**Table 2:** Primary Diagnosis

	Heparin group (*n* = 82)	Non-heparin group (*n* = 65)
STEMI	18 (22.0)	3 (4.6)
NSTEMI	11 (13.4)	3 (4.6)
Cardiogenic shock	16 (19.5)	9 (13.8)
CABG	12 (14.6)	37 (56.9)
Valve replacement	7 (8.4)	11 (16.9)
Tachyarrhythmias	9 (11.0)	0 (0)
Other	9 (11.0)	2 (3.1)

All values are n(%)

*STEMI* ST elevated myocardial infarction, *NSTEMI* non-ST elevated myocardial infarction, *CABG* coronary artery bypass graft

**Table 3:** Intra-Aortic Balloon Pump and Antithrombotic Characteristics

	Heparin group (*n* = 82)	Non-heparin group (*n* = 65)
Time on IABP (hours)^a^	42 (30–68)	26 (17–45)
Time in 1:1 Setting^a^	36 (18.8–62.5)	19 (10–40)
Time in 1:2 Setting^a^	5 (3–11)	4 (2–14)
Time in 1:3 Setting^a^	2 (1–3)	1
PTT goal range 60–80	54 (65.9)	N/A
PTT goal range 50–70	21 (25.6)	N/A
Other PTT goal	7 (8.5)	N/A
Rate of heparin (units/kg/hr)^a^	12 (9–15)	N/A
Time on heparin (hours)^a^	56 (32–89	N/A
Percent of time in therapeutic range^a^	44.8 ± 27.8	N/A
Received concomitant aspirin monotherapy	42 (51.2)	42 (64.6)
Received concomitant dual antiplatelet therapy^c^	22 (26.8)	9 (13.8)
Received concomitant dual antiplatelet therapy with clopidogrel and aspirin	14 (17.1)	9 (13.8)
Received concomitant dual antiplatelet therapy with ticagrelor and aspirin	8 (9.8)	0

All values are n(%) unless otherwise specified

*PTT* partial thromboplastin time

^a^mean +/−SD

^b^median (IQR)

^c^Dual antiplatelet therapy is defined as being on both aspirin and a P2Y_12_ inhibitor

The bleeding endpoint occurred in 17 (20.7%) heparin patients as compared to 13 (20.0%) non-heparin patients (*p* = 0.91) (Table 4). Of the patients in the heparin group who had a bleeding event, 9 patients had a PTT goal of 60–80 s, 3 had a PTT goal of 50–70 s, 3 patients had a PTT goal of 40–60, and 2 patients had a PTT goal of 50–60. There were no statistically significant differences in each individual component of the composite bleeding endpoint. The thrombotic endpoint occurred in 6 (7.3%) of patients in the heparin group as compared to 5 (7.7%) of patients in the non-heparin group (*p* = 0.1). There was no statistically significant difference in any of the individual components of this endpoint, including limb ischemia, device thrombosis, venous thromboembolism, or ischemic stroke. When the post-hoc logistic regression was performed for bleeding and thromboembolic events there were no significant differences found in the major composite outcomes (Table 4). There were no differences found in minor endpoints of hospital length of stay, intensive care unit length of stay, in-hospital morality, major outcome events happening within 24 h, or major outcome events occurring within 48 h of insertion (Table 5).

**Table 4:** Safety and Efficacy Outcomes

	Heparin group (*n* = 82)	Non-heparin group (*n* = 65)	Unadjusted *p* value	Adjusted* odds ratio (95% CI^a^)	Adjusted^*^ *p* value
Main bleeding endpoint	17 (20.7)	13 (20.0)	0.91	0.80 (0.31–2.09)	0.65
Major access site bleeding	2 (2.4)	3 (4.6)	0.47		
Minor access site bleeding	4 (4.9)	5 (7.7)	0.51		
Major non-access site bleeding	3 (3.7)	3 (4.7)	1		
Minor non-access site bleeding	7 (8.5)	1 (1.5)	0.08		
Main thrombotic endpoint	6 (7.3)	5 (7.7)	1	1.62 (0.42–6.3)	0.42
Limb ischemia	3 (3.7)	1 (1.5)	0.63		
Device thrombosis	1 (1.2)	0	N/A		
Venous thromboembolism	1 (1.2)	2 (3.1)	0.58		
Ischemic stroke	1 (1.2)	2 (3.1)	0.58		

All values are n(%)

^a^confidence interval

^*^A post hoc multivariate logistic regression was performed for major composite endpoints adjusting for sex, creatinine clearance, use of dual antiplatelet therapy, and patient’s location (coronary care unit versus cardiac surgery unit)

**Table 5:** Minor Outcomes

	Heparin group (*n* = 82)	Non-heparin group (*n* = 65)	*p* value
Hospital length of stay^a^	12 (7–23)	13.5 (9–21)	0.47
Intensive care unit length of stay^a^	5 (3–10)	5 (3–8)	0.50
In-hospital mortality	18 (22.0)	16 (24.6)	0.70
Bleeding and thrombotic events occurring within 24 h of placing IABP	11 (13.4)	7 (10.7)	0.24
Bleeding and thrombotic events occurring within 48 h of placing IABP	3 (3.7)	3 (4.7)	1

All values are n(%) unless otherwise specified

^a^median (IQR)

In the subgroup analysis of patients on outpatient anticoagulation prior to hospitalization, a total of 43 and 45 patients were included in the heparin and non-heparin groups, respectively. The bleeding endpoint occurred in 25.6 and 20% in the heparin and non-heparin groups, respectively (*p* = 0.40). The thrombotic endpoint occurred in 4.7 and 6.7% in the heparin and non-heparin groups, respectively (*p* = 1) (Table 6). There were no differences found in minor endpoints between groups (Table 7).

**Table 6:** Subgroup Analysis Major Outcomes

	Heparin group (*n* = 43)	Non-heparin group (*n* = 45)	*p* value
Composite endpoint of bleeding	11 (25.6)	8 (17.8)	0.44
Major access site bleeding	1 (2.3)	2 (4.4)	1
Minor access site bleeding	3 (6.9)	2 (4.4)	1
Major non-access site bleeding	2 (4.7)	3 (6.7)	1
Minor non-access site bleeding	5 (11.6)	1 (2.3)	0.12
Composite endpoint of thrombosis events	2 (4.7)	3 (6.7)	1
Limb ischemia	1 (2.3)	1 (2.2)	1
Device thrombosis	0	0	1
VTE	1 (2.3)	1 (2.2)	1
Ischemic stroke	0	1 (2.2)	1

All values are n(%)

**Table 7:** Subgroup Analysis Minor Outcomes

	Heparin group (*n* = 43)	Non-heparin group (*n* = 45)	*p* value
Hospital LOS	11 (6–16)^a^	12.5 (9–20)^a^	0.15
ICU LOS	5 (2.5–6)^a^	6 (3–7.5)^a^	0.91
In-hospital	9 (21)	8 (17.8)	0.71
Bleeding and thrombotic events occurring within 24 h of placing IABP	4 (9.3)	5 (11.1)	1
Bleeding and thrombotic events occurring within 48 h of placing IABP	3 (6.9)	2 (4.4)	1

All values are n(%) unless otherwise specified

^a^median (IQR)

## Discussion

In our retrospective analysis assessing the safety and efficacy of anticoagulation in patients on IABPs, we found no differences in major outcomes of bleeding or thrombotic events. These findings are similar to those of several other small studies comparing anticoagulation strategies in patients on an IABP [5–7].

Jiang et al. compared patients on an IABP with UFH versus those receiving no heparin in a single center, randomized trial. This study consisted of 153 patients who received either heparin with a goal aPTT of 50–70, or no anticoagulation while on an IABP. Patients in the heparin versus non-heparin group were matched based on demographics and comorbidities. As with our patients, they found no statistical differences in limb ischemia or IABP thrombus. Their study, however, did find statistically higher rates of bleeding in the heparin group as compared to the non-heparin group [6]. All patients in this group received either percutaneous coronary intervention or coronary artery bypass grafting, while our study included a more diverse patient population with any indication for an IABP, excluding endarterectomy.. Our study also looked at patients with different PTT goals, therefore providing additional information while in this study all heparinized patients had a PTT goal of 50–70.

Cooper et al. compared a selective heparin group (*n* = 102) versus a universally heparin group (*n* = 150) in a single center, prospective, cohort study. This study evaluated patients that had been admitted to the CCU. In the selective group, only patients with an indication for anticoagulation other than IABP received heparin. Similar to our analysis, this study found no statistical differences in limb ischemia, major IABP-related complications, or access site bleeding, though there was a statistically significant increase in overall bleeding events in the universal heparin group as compared to the selective heparin group. They also found no difference in CCU length of stay, total hospital length of stay, or mortality. Of the patients in the selective heparin group, 53% received heparin as compared to our analysis in which our control group received no anticoagulation [7].

Chin et al. retrospectively assessed 18,875 patients who received an IABP from 1996 to 2004 using data from the Benchmark Counterpulsation Outcomes Registry. This large observational study compared outcomes between patients who received anticoagulation as compared to those who did not receive anticoagulation. Overall, they found those who received anticoagulation had fewer in-hospital deaths and less limb ischemia without an increase in bleeding events. The authors concluded that anticoagulation should be used whenever possible for all IABP patients. This study differs from our findings and the findings of several retrospective studies demonstrating no difference in thrombotic outcomes when comparing anticoagulation strategies. Although this is a large study, it is difficult to draw any strong conclusions given the retrospective, observational nature of the study [9].

In patients on heparin who experienced any bleeding event, 8 had a PTT goal lower than 60–80 s. A lower PTT goal was likely targeted in these patients due to an increased bleeding risk at baseline. The last documented PTT was supratherapeutic in 6 patients with a major bleed. As previously mentioned, the heparin group had more patients on DAPT compared to the non-heparin group. This is most likely due to more patients in the heparin group presented with acute coronary syndrome events. Of those who had a bleeding event, 6 (37.5%) were receiving concomitant DAPT in the heparin group, and 2 (16.7%) were on concomitant DAPT in the non-heparin group. Although DAPT has been proven to increase bleed risk, especially in combination with anticoagulation, there was still no difference in bleeding shown in our study in our post hoc multivariate analysis adjusting for DAPT [10, 11].

There were several limitations to this study. The majority of patients in the heparin group were medical patients admitted to the CCU, while the majority of non-heparinized patients were surgical patients admitted to the CSICU. Cardiac surgery patients may have a higher baseline risk of bleeding post operatively than medical patients despite use of anticoagulation. Rates of reoperation due to bleeding can be up to 8% post CABG, and there were significantly more patients in the non-heparin group who received CABG [12]. Of the patients who experienced a bleed, 5 (32.2%) patients in the heparin group were cardiac surgery patients and 8 (66.7%) patients in the non-heparin group were cardiac surgery patients. Despite these potential differences in patient populations, there was no difference found in hospital length of stay, ICU length of stay, or mortality between the two groups. It is also important to note that the heparin group may have been more susceptible to IABP related adverse events given that they were on an IABP for longer. Our subgroup analysis also has limitations. We attempted to examine a patient population with no other indication for anticoagulation by excluding patients who had an outpatient prescription for an anticoagulant prior to admission. If there was no documentation of an outpatient prescription, or if the patient had a new event requiring anticoagulation (i.e. new onset atrial fibrillation during indexed hospitalization), they were not excluded from this subgroup analysis. Another limitation of this study is that it does not explore patients who were on anticoagulants other than heparin (i.e. bivalirudin). We excluded these patients in order to directly compare heparin to no anticoagulation, as heparin is the preferred agent at our institution due to factors such as ability to reverse, lack of renal clearance, and cost. Patients were also excluded if they underwent an endarterectomy procedure, as there is a very specific, fixed rate of heparin utilized by our CSICU service based on chest tube output.

There are no large, prospective, randomized, controlled trials comparing anticoagulation strategies in IABPs. Although this is a small, single center study, our study would be the first to compare anticoagulation versus non-anticoagulation in patients on an IABP within the past 10 years.

Overall, our study had similar rates of bleeding and thrombotic events when compared to other literature surrounding IABP use [2, 3, 5–8]. Although we did not find a statistically significant difference in bleeding, there were numerically more reported bleeding events in the heparin group as compared to the non-heparin group.

## Conclusion

There were no significant differences found in major endpoints of bleeding and thrombotic events in patients who received anticoagulation while on an IABP versus those who did not receive anticoagulation. Further evaluation of the benefit of anticoagulation in IABPs needs to be explored in larger, prospective trials.

## Data Availability

Not applicable.
